# A Case of Perforated Sigmoid Diverticulitis in Which Gram Staining of Ascitic Fluid Was Useful for Diagnosis

**DOI:** 10.1155/2014/417987

**Published:** 2014-03-04

**Authors:** Junko Tsuchida, Shouhei Fujita, Fumihiro Kawano, Ryoichi Tsukamoto, Kunpei Honjo, Shigetoshi Naito, Shun Ishiyama, Shozo Miyano, Michio Machida, Toshiaki Kitabatake, Minoru Fujisawa, Kuniaki Kojima, Kanako Ogura, Toshiharu Matsumoto

**Affiliations:** ^1^Department of General Surgery, Juntendo University Nerima Hospital, 3-1-10 Takanodai, Nerimaku, Tokyo 177-8521, Japan; ^2^Department of Clinical Pathology, Juntendo University Nerima Hospital, 3-1-10 Takanodai, Nerimaku, Tokyo 177-8521, Japan

## Abstract

An 85-year-old woman was admitted to our hospital for steroid therapy for relapsing nephrotic syndrome. During hospitalization, she complained of sudden epigastric pain at night. Although there were signs of peritoneal irritation, CT showed a large amount of ascitic fluid, but no free intraperitoneal gas. Gram staining of ascitic fluid obtained by abdominal paracentesis showed Gram-negative rods, which raised a strong suspicion of gastrointestinal perforation and peritonitis. Therefore, emergency surgery was performed. Exploration of the colon showed multiple sigmoid diverticula, one of which was perforated. The patient underwent an emergency Hartmann's procedure. Imaging studies failed to reveal any evidence of gastrointestinal perforation, presenting a diagnostic challenge. However, a physician performed rapid Gram staining of ascitic fluid at night when laboratory technicians were absent, had a strong suspicion of gastrointestinal perforation, and performed emergency surgery. Gram staining is superior in rapidity, and ascitic fluid Gram staining can aid in diagnosis, suggesting that it should be actively performed. We report this case, with a review of the literature on the significance of rapid diagnosis by Gram staining.

## 1. Introduction

The presence of free intraperitoneal air (or pneumoperitoneum) is an important finding in the diagnosis of gastrointestinal perforation. However, pneumoperitoneum is not always associated with gastrointestinal perforation. In the absence of free intraperitoneal air, whether or not to perform surgery must be determined based on abdominal physical findings or other diagnostic means. In particular, the prognosis of patients with large-bowel perforation reportedly depends on the time to surgery, necessitating rapid diagnosis.

Herein, we report a case of sigmoid colon perforation diagnosed by rapid, very simple Gram staining of ascitic fluid, with a review of the literature.

## 2. Case Report

The patient was an 85-year-old woman with a chief complaint of epigastric pain and a past history of nephrotic syndrome, hyperlipidemia, diabetes mellitus, and status postbilateral cataract surgery. In 2009, she developed nephrotic syndrome, which was treated with steroid therapy until February 2011, when complete remission was achieved and the therapy was discontinued. No kidney biopsy was performed because of her advanced age, and the cause of her nephrotic syndrome remained unknown. In February 2013, the nephrotic syndrome relapsed, and she was admitted to the Department of Nephrology of our hospital for steroid therapy. Steroid semipulse therapy (500 mg of methylprednisolone daily for 3 days) and posttherapy (prednisolone tapering from 30 mg daily) were initiated. During admission, she developed sudden epigastric pain and was referred to our department for consultation.

**Figure 1:**
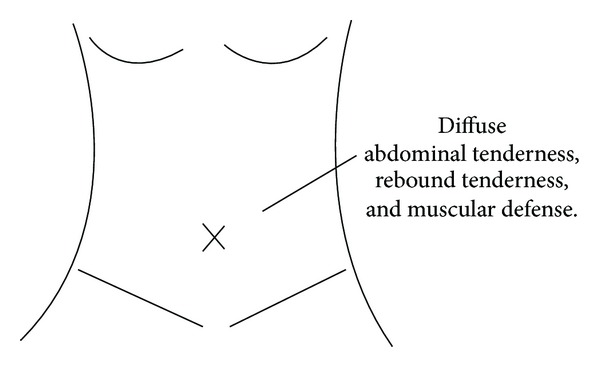


She was 147 cm tall and weighed 44 kg, with a physical status of ASA 3. Her heart rate was 108/min, blood pressure 158/92 mmHg, and respiratory rate 15/min, with an SpO_2_ of 95%. There was diffuse abdominal tenderness, rebound tenderness, and muscular defense (see Figure 1 and Table 1).

### 2.1. Plain Abdominal X-Ray Film (Supine)

**Figure 2:**
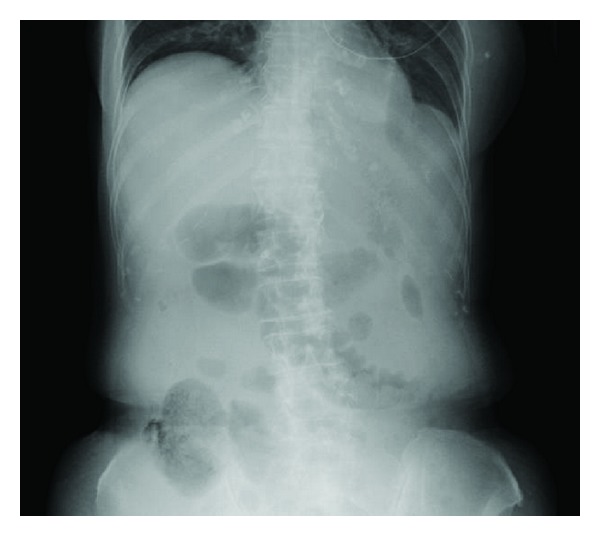
Plain abdominal X-ray film. No free intraperitoneal gas was observed.

Bowel gas was deviated toward the midline, suggesting ascites. No free intraperitoneal gas was noted (Figure 2).

### 2.2. Plain Abdominal CT

**Figure 3:**
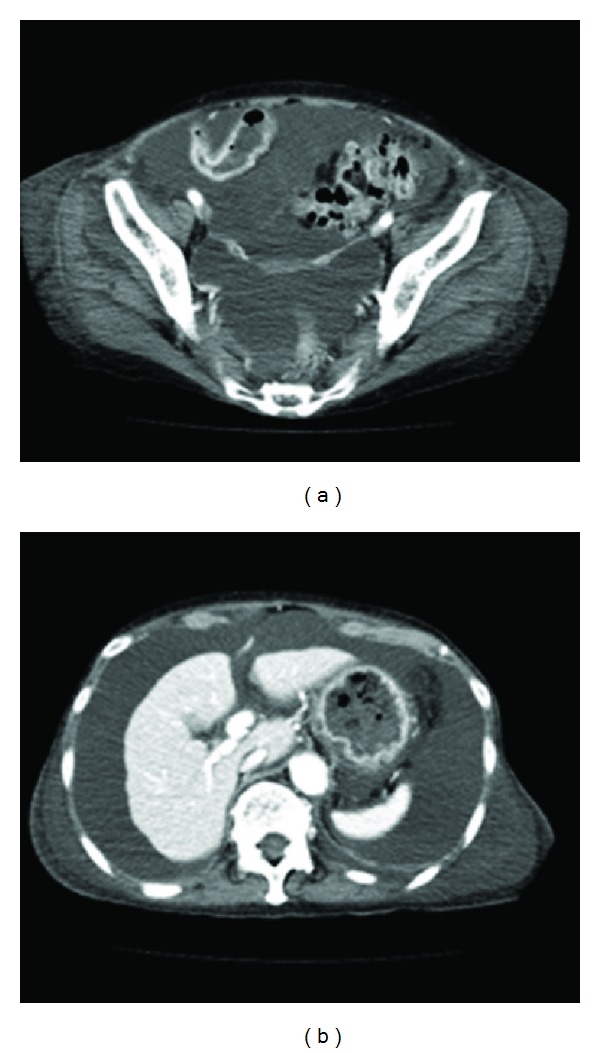
Plain abdominal CT. (a) Multiple sigmoid diverticula. (b) A large amount of ascitic fluid. No evidence of perforation was found.

Multiple sigmoid diverticula and a large amount of ascitic fluid were observed, with no evidence of bowel perforation (Figure 3).

This patient was an elderly woman who had nephrotic syndrome as an underlying condition and was taking steroids. Imaging studies failed to reveal any evidence of gastrointestinal perforation, presenting a diagnostic challenge as to whether or not to perform emergency surgery. Therefore, we performed abdominal paracentesis in the presence of a large amount of ascitic fluid.

### 2.3. Ascitic Fluid

**Figure 4:**
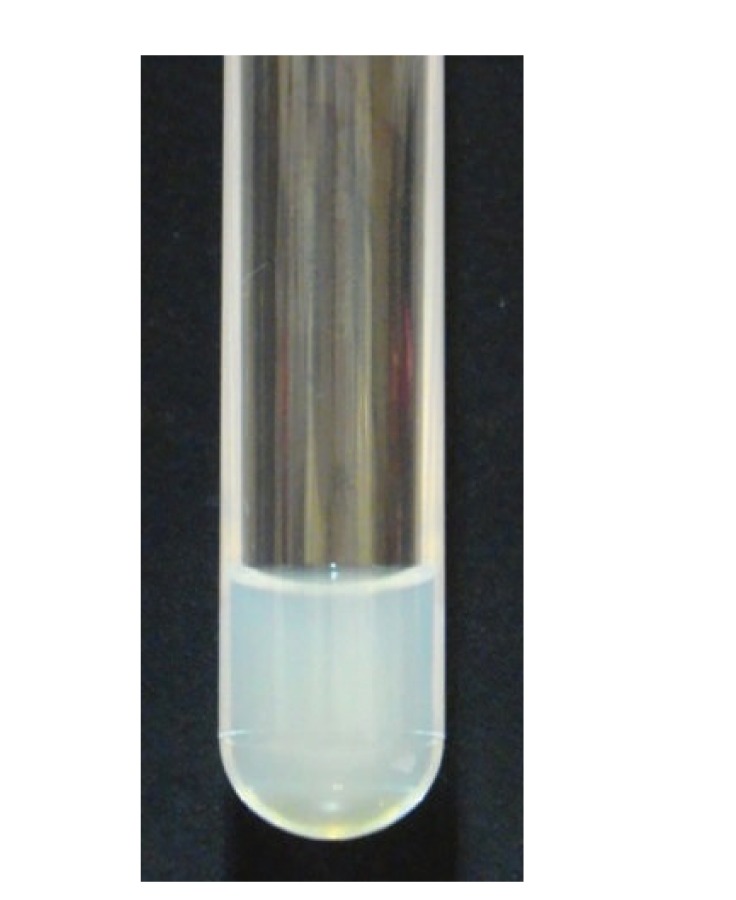
Ascitic fluid was milky, clear, and odorless.

The ascitic fluid was milky, clear, odorless, with a protein content of 0.3 g/dL, an LDH level of 488 IU/L, and a specific gravity of 1.010 (Figure 4).

Since the ascitic fluid had no smell of feces or that of the digestive system and was transudative, we considered that the presence of intestinal bacteria in the ascitic fluid, which should normally be sterile, would raise the possibility of gastrointestinal perforation. In addition, since it was at night when laboratory technicians were absent, a physician performed Gram staining.

### 2.4. Gram Staining

**Figure 5:**
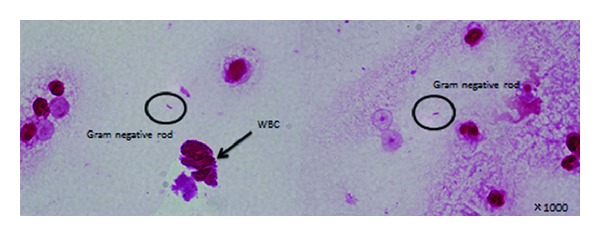
Rapid Gram staining of ascitic fluid. A small number of Gram-negative rods and a large number of leukocytes were seen.

A small number of Gram-negative rods and a large number of leukocytes were seen (Figure 5).

Since Gram staining showed the presence of Gram-negative rods in the ascitic fluid, we strongly suspected digestive tract perforation and performed emergency surgery.

### 2.5. Intraoperative Findings

The abdomen was opened through a long midline abdominal incision. Ascitic fluid had accumulated in the peritoneal cavity. There were multiple sigmoid diverticula, one of which was perforated, and so Hartmann's procedure was performed with a diagnosis of perforated sigmoid diverticulum.

The operative time was 2 h and 43 min. The amount of blood loss was 160 mL. Two units of MAP, 10 units of PCs, and 2 units of FFP were transfused.

### 2.6. Resected Specimen

**Figure 6:**
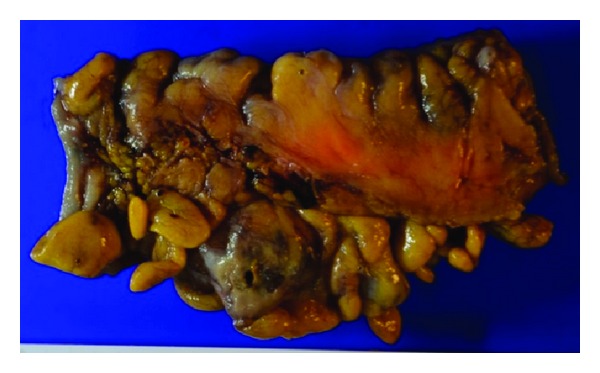
Resected specimen of the sigmoid colon. Multiple stool-filled diverticula were found, one of which had a perforation, through which stool was visible.

The resected sigmoid colon specimen measured 10 × 8 cm and showed multiple stool-filled diverticula, one of which had a perforation, through which stool was visible (Figure 6).

### 2.7. Histopathological Findings

**Figure 7:**
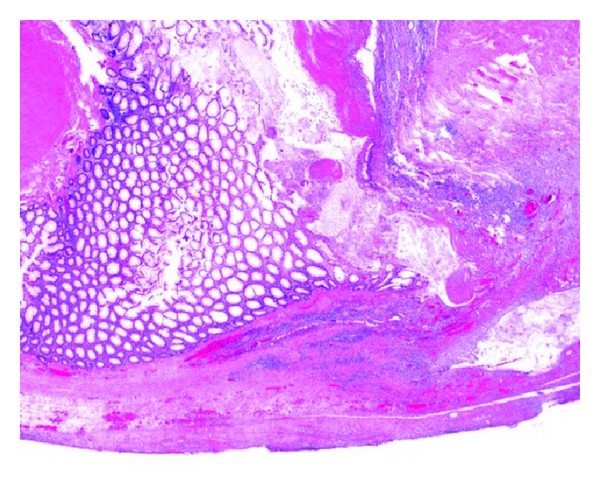
Histopathological findings. Diverticular perforation with neutrophilic infiltration of the surrounding tissue.

Multiple diverticula extending into the subserosa were present, and one of them was perforated. At this site, the diverticular wall was disrupted, and the surrounding tissue was infiltrated by neutrophils (Figure 7).

### 2.8. Microbiological Examinations of Ascitic Fluid

At a later date, the results of microbiological examinations of ascitic fluid were reported by the laboratory technician. Gram-stained smears showed a small number of Gram-negative rods, Gram-positive cocci and rods, and +2 leukocytes. Cultures were positive for intestinal bacteria, including Bacteroides fragilis, being consistent with the results of the rapid diagnosis by Gram staining (Table 2).

### 2.9. Postoperative Course

The postoperative course was uneventful, and the patient was transferred to the general ward. Later, her hospital course became complicated by a urinary tract infection, but she was discharged, after rehabilitation, on the 64th postoperative day.

## 3. Discussion

In the absence of imaging findings suggestive of gastrointestinal perforation, this patient presented a diagnostic challenge. However, since rapid Gram staining of ascitic fluid revealed Gram-negative rods, we strongly suspected gastrointestinal perforation, performed emergency surgery, and found a sigmoid colon perforation. Although it was at night, we performed rapid Gram staining, strongly suspected a gastrointestinal perforation, and were able to treat it surgically at an early stage.

Colon perforation easily causes septic shock due to intestinal bacteria, which leads to multiorgan failure with a poor prognosis if management is delayed. It is an important disease with a reported mortality rate of 17.6–29.4% [1–4]. Kuroda et al. [1] listed the following as prognostic factors for gastrointestinal perforation: (1) age ≥ 70 years, (2) preoperative development of septic shock as a complication, (3) WBC ≤ 4,000/mm^3^, (4) BE ≤ −5, (5) APACHE II score ≥ 20, (6) generalized peritonitis, and (7) time to surgery ≥24 hours. The time from onset to surgery is an important factor influencing the patient's prognosis. Therefore, it is necessary to make an early diagnosis of this condition using simple, rapid tests and start treatment.

Several radiologic modalities have a role in the diagnosis of gastrointestinal perforation, including plain X-ray film, ultrasonography, and computerized tomography [5]. CT is minimally invasive, and provides many objective data on the entire intraperitoneal contents within a short period of time [6, 7]. It has been reported that the presence of free intraperitoneal air on CT indicates gastrointestinal perforation with a probability of 98.7% [8]. However, despite the presence of gastrointestinal perforation, as in the present case, CT did not reveal the presence of free intraperitoneal air in 25% [8], 83.3% [9], 64.3% [10], and 80.5% [11] of cases. In such cases, a decision must be made as to whether or not to perform surgery based on physical examination of the abdomen and other diagnostic means.

Gram staining can determine whether the patient has an infection within about 30 minutes after specimen collection. It is a very inexpensive test that can be performed using a glass slide and a small amount of staining solution [12], can estimate microorganisms based on their morphological characteristics, and is useful for the choice of subsequent treatments. In particular, if microorganisms are detected in normally sterile body fluids, such as blood, cerebrospinal fluid, pleural effusion, ascitic fluid, and joint fluid, they can be determined as pathogenic bacteria [12]. On the other hand, the disadvantage of Gram staining is the failure to detect a small number of microorganisms in a specimen: it is said that the specimen must contain more than 100,000 bacteria/mL to be deemed Gram positive. It is reportedly possible to microscopically examine as few as 1,000–10,000 bacteria/mL of cerebrospinal, ascitic, or pleural fluid aspirate after centrifugation or sedimentation. Therefore, aspirates should be subjected to centrifugation or sedimentation before Gram staining. In addition, the results may vary according to the proficiency of the examiner, leading to a possible misdiagnosis [13]. For this reason, physicians should practice staining techniques and receive training in microscopy to aid in making an urgent diagnosis by Gram staining.

## 4. Conclusion

In this patient, CT findings were insufficient to suspect gastrointestinal perforation, but the detection of Gram-negative rods by Gram staining prompted us to perform emergency surgery. Gram staining should be performed in patients whose imaging shows no evidence of gastrointestinal perforation. If physicians themselves perform Gram staining, it may lead to prompt therapeutic intervention, making Gram staining an examination technique to be actively learned.

## Figures and Tables

**Table 1a:** (a)

Complete blood count	
WBC	2.90 × 10^9^/L
RBC	4.20 × 10^12^/L
Hb	13.2 g/dL
Hct	39.6%
Plt	120 × 10^9^/L

**Table 1b:** (b)

Biochemistry	
TP	4.6 g/dL
Tbil	0.7 mg/dL
AST	16 IU/L
ALT	22 IU/L
CK	40 IU/L
Amy	33 IU/L
BUN	24 mg/dL
Cre	0.84 mg/dL
Na	137 mmol/L
K	4.0 mmol/L
CRP	19.91 mg/dL

**Table 2a:** (a) Microbial smear examination

Name of bacteria	Number of bacterial cells
Gram-negative rods	Small
Gram-positive cocci	Small
Gram-positive rods	Small
Leukocytes	2+

**Table 2b:** (b) Bacterial cultures

Name of bacteria	Number of bacterial cells
*Bacteroides fragilis* group	1+
*Propionibacterium* sp.	Small
*Micromonas * micros	Small
Alpha-streptococci	Small
*Bacillus* sp.	Small
